# Epigenetic Silencing of CXCR4 Promotes Loss of Cell Adhesion in Cervical Cancer

**DOI:** 10.1155/2014/581403

**Published:** 2014-07-10

**Authors:** Suresh Singh Yadav, Shyam Babu Prasad, Mitali Das, Soni Kumari, Lakshmi Kant Pandey, Sunita Singh, Satyajit Pradhan, Gopeshwar Narayan

**Affiliations:** ^1^Department of Molecular and Human Genetics, Banaras Hindu University, Varanasi 221005, India; ^2^Department of Obstetrics and Gynecology, Banaras Hindu University, Varanasi 221005, India; ^3^Department of Zoology, Mahila Mahavidyalaya, Banaras Hindu University, Varanasi 221005, India; ^4^Department of Radiotherapy & Radiation Medicine, Banaras Hindu University, Varanasi 221005, India

## Abstract

In the network of chemokine signaling pathways, recent reports have described the SDF-1*α*/CXCR4 axis and its role in cancer progression and metastasis. Interestingly, we found downregulation of CXCR4 at both transcript and protein level in cervical cancer cell lines and primary tumors. We also found CXCR4 promoter hypermethylation in cervical cancer cell lines and primary biopsy samples. DNA hypomethylating drug 5-AZA-2′-deoxycytidine and histone deacetylase inhibitor Trichostatin A treatments in cell lines reactivate both CXCR4 transcription and protein expression. Cell adhesion assay demonstrated that autocrine SDF-1*α* promotes the loss of cell adhesion while paracrine SDF-1*α* predominantly protects the normal cervical cells from loss of cell adhesion. Cervical cancer cell line C-33A having increased expression of CXCR4 after TSA treatment showed increased cell adhesion by paracrine source of SDF-1*α* in comparison to untreated C-33A. These findings demonstrate the first evidence that epigenetic silencing of CXCR4 makes the cells inefficient to respond to the paracrine source of SDF-1*α* leading to loss of cell adhesion, one of the key events in metastases and progression of the disease. Our results provide novel insight of SDF-1*α*/CXCR4 signaling in tumor microenvironment which may be promising to further delineate molecular mechanism of cervical carcinogenesis.

## 1. Introduction

The process of cancer progression and metastasis, driven by chemokines and its receptors, is a major cause of death in cancer patients [1, 2]. Chemokines are a family of chemoattractive cytokines and are grouped into C, CC, CXC, and CX3C subfamilies based on the position of conserved cysteine residues [3, 4]. Chemokine receptors are a family of seven transmembrane G protein-coupled cell surface receptors (GPCRs) and are defined by their ability to induce directional migration of cells towards a gradient of chemokine (chemotaxis). These receptors, initially identified on leukocytes, are present on many different cell types. It has been demonstrated that hematopoietic and nonhematopoietic cells express these receptors for various chemokines that are constitutively expressed in tumor microenvironments [5]. The cross talk between cancer cells and their microenvironment has an important influence on tissue homeostasis and progression of cancers [6]. Chemokines are the major mediators of this cross talk between tumor cell and stroma [7]. The interactions between chemokine receptors and their respective chemokines help to coordinate the tumor cell growth and trafficking [8].

Among chemokine signaling axes, the SDF-1*α*/CXCR4 has been demonstrated to have a critical role in various solid tumors [9]. CXCR4 is a G protein-coupled transmembrane receptor initially identified as a cofactor for HIV entry into CD4^+^ T cells [10]. Activation of CXCR4 with SDF-1*α* triggers G protein signaling that activates a variety of intracellular signal transduction pathways and molecules regulating migration, chemotaxis, cell survival, proliferation, and adhesion [11–13]. Involvement of SDF-1*α*/CXCR4 in cancer progression is increasingly being appreciated. It has been demonstrated that SDF-1*α* enhances the chemotaxis, chemoinvasion, and adhesive properties of breast cancer cells, parameters that are critical for development of metastasis [14]. Orimo et al. [15] have shown that stromal fibroblasts present in invasive human breast carcinoma promote tumor growth through elevated SDF-1*α* secretion. Exploring the autocrine and paracrine signaling, Tsujikawa et al. [16] have demonstrated that chemokine CCL22 produced by cancer cells themselves (autocrine) or by other types of cells, for example, macrophage (paracrine), increased the cell motility of CCR4^+^ head and neck squamous cell carcinoma cells* in vitro*. CXCR4 has been shown to be upregulated in many of the solid tumors including cervical cancer (CC) [17, 18]. Also, downregulation of CXCR4 has been demonstrated in pancreatic cancer cell [19] and CD34^+^ cells [20] of patients with primary myelofibrosis due to promoter hypermethylation. Downregulation of SDF-1*α* also has been reported in colonic carcinoma [21] and human astrocytoma [22]. In continuation with these reports, Nikkhoo et al. [23] have demonstrated recently that nuclear expression CXCR4 is associated with a better overall survival of patients with gastric cancer. These literatures regarding CXCR4 indicate that CXCR4 signaling is not limited to promote tumor progression only; it is also involved in maintaining normal homeostasis of cells/tissue.

Little is known about the transcriptional regulation of CXCR4 and its importance in tumor microenvironment. Source of SDF-1*α* (autocrine or paracrine) and its interaction with CXCR4 may determine further signaling and its role in cancer progression. Expression analysis of CXCR4 in all CC cell lines has not been studied yet; hence, we thought to study CXCR4 expression in CC cell lines. In this study, we have explored the interaction of CXCR4 with the paracrine and autocrine source of SDF-1*α*. This study demonstrates epigenetic silencing of CXCR4 in CC cell lines and tumor biopsy samples by promoter hypermethylation and histone deacetylation. Interestingly, our data also demonstrates that epigenetic silencing of CXCR4 in CC cell lines leads to loss of cell adhesion, one of the key events in metastases and cancer progression. Targeting SDF-1*α*/CXCR4 signaling could have therapeutic and prognostic implications. This is the first report which depicts the antitumor activity of SDF-1*α*/CXCR4 signaling in CC.

## 2. Materials and Methods

### 2.1. Primary Biopsy Samples and Cervical Cancer Cell Lines

Normal cervical tissues (*N* = 30), primary tumor biopsy samples (*n* = 63), and their clinical information were collected as per protocol approved by the institutional ethical committee after patient's written informed consent. Normal cervical tissues were taken from the noninflamed epithelial layer of ectocervix of patients undergoing hysterectomy due to either fibroid (*N* = 18) or prolapsed (*N* = 12) uterus. Ectocervix is the part of cervix which has squamous lining (glandular elements are present in the endocervix and at the squamocolumnar junction). Histology of normal samples and inflammation status was further confirmed by hematoxylin-eosin staining of tissue sections and samples having inflammation and glandular epithelium were excluded from study. Patients for normal biopsy were with mean age of 47 years (age range 39–60 years) and for cervical cancer patients were with mean age of 49 years (age range 30–70 years). Tissues were either stored in RNAlater (Ambion, USA) at −20°C or immediately used for RNA or protein isolation. Total of eight CC cell lines (HeLa, SiHa, ME-180, C-33A, CaSki, C-4I, MS751, and SW756) that have been previously characterized [24–27] were kind gift from Dr. V. V. S. Murty, Columbia University, New York, USA. HEK293 cell line was purchased from National Center for Cell Science (NCCS), Pune, India. Two normal cervical tissues from two different patients (NC65 and NC66) were cultured in complete RPMI1640 media. All cell lines were maintained in recommended culture media supplemented with 10% fetal bovine serum (GIBCO, USA), streptomycin, and penicillin at 37°C in a humidified atmosphere containing 5% CO_2_.

### 2.2. Reverse Transcriptase PCR

Total RNA was isolated from tissue samples and cell lines samples using TRizol (Invitrogen, USA), following the manufacturer's protocol followed by DNaseI (Fermentas, USA) treatment. Purified RNA was stored at –80°C. The total RNA was quantified by NanoDrop (Thermo Scientific, USA). The first strand cDNA synthesis was performed using high capacity cDNAreverse transcription kit (ABI, USA) according to the manufacturer's protocol. Semiquantitative RT-PCR was performed using CXCR4 and internal control *β*-Actin primers (see Supplementary Table S1 in Supplementary Material available online at http://dx.doi.org/10.1155/2014/581403) in normal cervix, CC cell lines, and tumor biopsy samples

To validate the semiquantitative PCR results, quantitative real time RT-PCR was performed with 2X SYBR Green PCR master mix (ABI, USA) according to the manufacturer's protocol using CXCR4 and *β*-Actin (as an internal control) expression primers (Supplementary Table S1). Briefly, 12.5 *μ*L SYBR Green PCR master mix, 4 pM of each primer, and 50 ng of cDNA were used to determine the mRNA expression levels of CXCR4 by the real time PCR (Applied Biosystems). PCR was performed in duplicate for all the samples for both CXCR4 and internal control and was repeated at least twice. Relative gene expression of CXCR4 and *β*-Actin was analyzed using comparative C_T_ method [28].

### 2.3. Sodium Bisulphite Treatment and Methylation Specific PCR (MSP) and Bisulphite Sequencing

Genomic DNA was isolated according to a standard proteinase K digestion and phenol/chloroform extraction protocol [29]. For DNA methylation analysis, sodium bisulfite modification of genomic DNA was performed using EpiTect Bisulfite Kit (Qiagen GmbH, Germany). Bisulfite-modified DNA was amplified with primer sets specific for methylated or unmethylated sequences (Supplementary Table S1). The PCR for all samples demonstrating promoter hypermethylation of CXCR4 was repeated to confirm these results. To validate the MSP results and to confirm the methylation status, bisulphite sequencing was done in four CC cell lines (SiHa, SW756, MS751, and C-33A).

### 2.4. Drug Treatment of Cell Lines

We treated six CC cell lines (HeLa, SiHa, ME-180, C-33A, MS751, and SW756) with the DNMT (DNA methyltransferase) inhibitor 5-AZA-2′-deoxycytidine (AZA) (5 *μ*M for 5 days) and/or HDAC (histone deacetylase) inhibitor Trichostatin A (TSA) (200 nM for 24 hrs) as previously described [27]. For cell adhesion assay conditioned media (spent media harvested from 50 to 60% confluent cultured cells) were used and the cells were incubated with AMD3100 (500 ng/mL), an inhibitor of SDF-1*α*/CXCR4 signaling [30, 31], for 10 minutes prior to cell adhesion.

### 2.5. Immunoblotting

The whole cell proteins were isolated by lysing tissue samples stored at −80°C and CC cell lines in RIPA buffer [100 mM NaCl, 50 mM Tris-Cl (pH 7.4), 2 mM EGTA, 1 mM EDTA, 1 mM DTT, 1 mM PMSF, 1% NP-40, and 0.1% SDS] containing protease inhibitor cocktail (Sigma, USA) on ice. Protein concentration was measured by the Bradford assay. Equal amount of protein (50 *μ*g) was separated using 10% SDS-PAGE and transferred to PVDF membrane (Millipore Corporation, Billerica, MA, USA). Membrane was blocked with 5% nonfat milk in TBST buffer (Tris-Cl, NaCl, Tween-20) for one hour at room temperature and probed with rabbit anti-CXCR4 primary antibody (Abcam, USA) in 5% nonfat milk in TBST overnight at 4°C. After washing with TBST, membrane was incubated with ALP-conjugated goat anti-rabbit IgG (Bangalore Genie, India) in 5% nonfat milk in TBST. After washing twice with TBST, the blot was developed with NBT/BCIP solution (Amresco, USA) and imaged. The membrane was stripped and reprobed with anti-GAPDH antibody (Imgenex, India) as a loading control. All of the immunoblot experiment was repeated twice.

### 2.6. Immunohistochemistry

Immunohistochemistry was done on paraffin embedded tissue sections. Rabbit polyclonal anti-CXCR4 primary antibody (Abcam, USA) was used in 1 : 100 dilutions. Peroxidase conjugated anti-rabbit secondary antibody was used against the primary antibody. Chromogenic detection was done by using 3,3′-diaminobenzidine (DAB) as substrate for peroxidase. Slides were counterstained with hematoxylin. Brown stains were considered as positively stained for CXCR4. Nuclei were stained by blue colour of hematoxylin.

### 2.7. Enzyme-Linked Immunosorbent Assay (ELISA)

ELISA was done to measure the concentration of SDF-1*α* protein in conditioned media of NC65, NC66, and HEK293. The culture supernatant was harvested and centrifuged to remove cellular debris, and Human CXCL12 ELISA kit (R&D Systems, UK) was used to measure chemokine production in each supernatant according to the manufacturer's instructions. The ELISA assay was carried out twice in duplicate.

### 2.8. Cell Adhesion Assay

Cell adhesion assays were performed using Vybrant Cell Adhesion Assay Kit (Molecular Probes) in fibronectin (7 *μ*g/cm^2^) coated 96-well culture plates as per recommended protocol. For all the sets of experiments, cells used were in similar growth condition (semiconfluent). Conditioned media of cells (NC65, NC66, and HEK293) having the expression of SDF-1*α* were used as paracrine source of SDF-1*α*. Briefly, cells were harvested, washed with PBS, and resuspended in incomplete media with 5 *μ*M calcein AM dye. Cells were washed and resuspended in different concentration of recombinant SDF-1*α* or in conditioned media with or without AMD3100, followed by seeding the cells in fibronectin coated 96-well culture plates in triplicate with cell density ~5 × 10^4^ cells/well. Cells were incubated for 15 minutes in optimum culture conditions for cell adhesion. Nonadherent cells were washed properly with PBS and adherent cells were quantified by fluorescence measurement by fluorescence plate reader at 494 ± 20 nm wavelength. All the experiments were performed thrice independently in triplicate. Results were presented as percentage cell adhesion of total cells seeded.

### 2.9. Flow Cytometry

For evaluation of the surface expression of CXCR4, flow cytometry was done after AZA and TSA treatments. After the treatment, cells were harvested, followed by labeling with antibody. Briefly, harvested cells were washed in ice cold PBS and resuspended in PBS having 1% FBS and 1% sodium azide at cell density of 1 × 10^6^ cells/mL in Eppendorf tubes. Cells were incubated with rabbit anti-human CXCR4 primary antibody (Abcam, USA) for 30 minutes at room temperature, followed by washing and incubation with FITC labelled goat anti-rabbit IgG (Merck Genei, India) for 30 minutes at room temperature. After washing in ice cold PBS, cells were fixed in 1% paraformaldehyde. Surface expression of CXCR4 was analyzed using BD FACSCalibur flow cytometer (BD Biosciences, USA) in FL1-H channel.

### 2.10. Statistical Analysis

Expression of mRNA levels is presented as mean ± SD. Normalized expression values less than [mean of normal − SD] were considered to be downregulated. Data of cell adhesion were expressed as mean ± SEM. %cell adhesion was calculated by formula (adherent cells × 100)/total cells. One way ANOVA followed by the Bonferroni multiple comparison post hoc test was performed for comparisons between more than two groups. Paired 2-tailed Student's *t*-test was applied for comparisons between two groups. GraphPad Prism5 software (La Jolla, USA) was used for analysis. *P* value less than 0.05 was considered to be statistically significant.

## 3. Results

### 3.1. Constitutive CXCR4 Expression Is Frequently Downregulated

**Figure 1:**
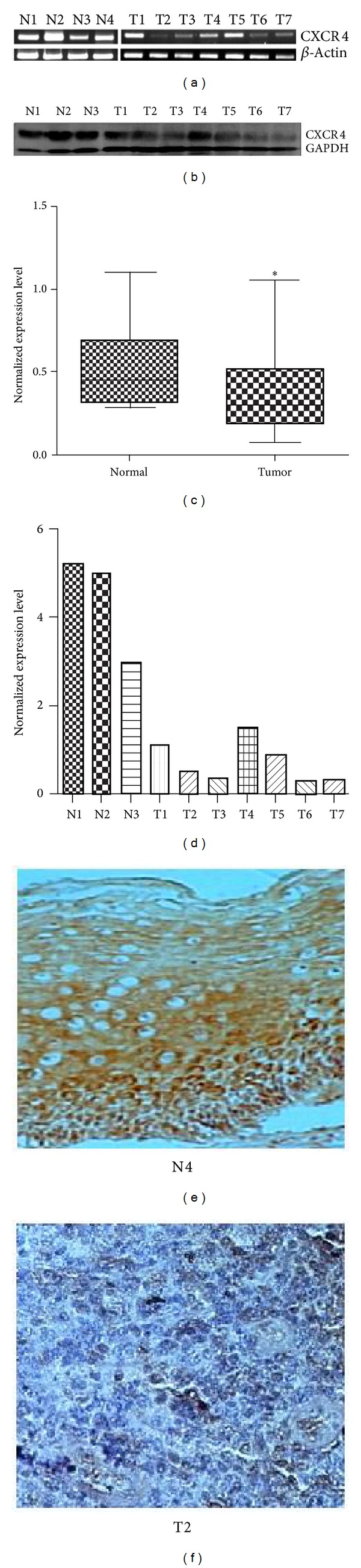
Expression of CXCR4. (a) Semiquantitative reverse transcriptase PCR in normal cervix and tumor biopsy samples. Upper panel is CXCR4 and lower panel is *β*-Actin. (b) Immunoblotting with the cell lysate of normal cervix and tumor biopsy. (c) Box plot showing significant downregulation of CXCR4 in primary tumor (*P* = 0.007). (d) Histogram showing the result of immunoblotting in normal cervix and tumor biopsy. (e) Immunohistochemistry showing expression of CXCR4 in normal squamous epithelial cells. (f) Immunohistochemistry showing expression of CXCR4 in tumor biopsy sample. *P value ≤ 0.05. N = normal cervix and T = tumor biopsy.

Expression profiling of CXCR4 transcript was done by semiquantitative RT-PCR in eight CC cell lines (HeLa, SiHa, ME-180, C-33A, CaSki, C-4I, MS751, and SW756), 63 (60 squamous cell carcinomas and 3 adenosquamous) primary tumor biopsies, and 30 normal cervical tissue samples. Our data showed significant downregulated transcription of CXCR4 in 32 out of 63 (51%) primary tumor biopsies (*P* = 0.007) relative to normal cervical tissues (*n* = 30) (Figures 1(a) and 1(c)). Semiquantitative RT-PCR result was validated by quantitative real time PCR which showed 1.4–68.5-fold downregulation of CXCR4 in primary tumor (representative data in Supplementary Table S2). Expression pattern of CXCR4 at protein level was also investigated by immunoblotting (Figure 1(b)). Concordant with the transcript expression pattern, 55% (*n* = 20) tumor showed downregulated expression of CXCR4 (Figure 1(d)). All the cell lines tested except HeLa (88%, *n* = 8) showed little or nil expression of CXCR4 at transcription level (Figure 3(a) upper panel). To see the expression of CXCR4 at protein level in cell lines, we also did the immunoblotting using SiHa, C-33A, and SW756. Concordant with our RT-PCR results, immunoblots also showed similar results (Figure 3(a) lower panel). To show the expression of CXCR4 in squamous epithelial cell of normal cervical tissue, we did the immunohistochemistry in five normal and eight tumor biopsy samples. Concordant with RT PCT and immunoblotting, CXCR4 was expressed in normal cervical squamous epithelial cell which was differentially downregulated in tumor tissue (Figures 1(e) and 1(f)). We analyzed the clinical correlation of CXCR4 expression with tumor size, lesion type (proliferative, ulceroproliferative, infiltrative, ulceroinfiltrative, and ulcerative), tumor stage (IIA, IB, IIB, and IIIB), and grade (poorly differentiated and undifferentiated), but we did not find significant difference among the groups.

### 3.2. CXCR4 Promoter Is Frequently Hypermethylated

**Figure 2:**
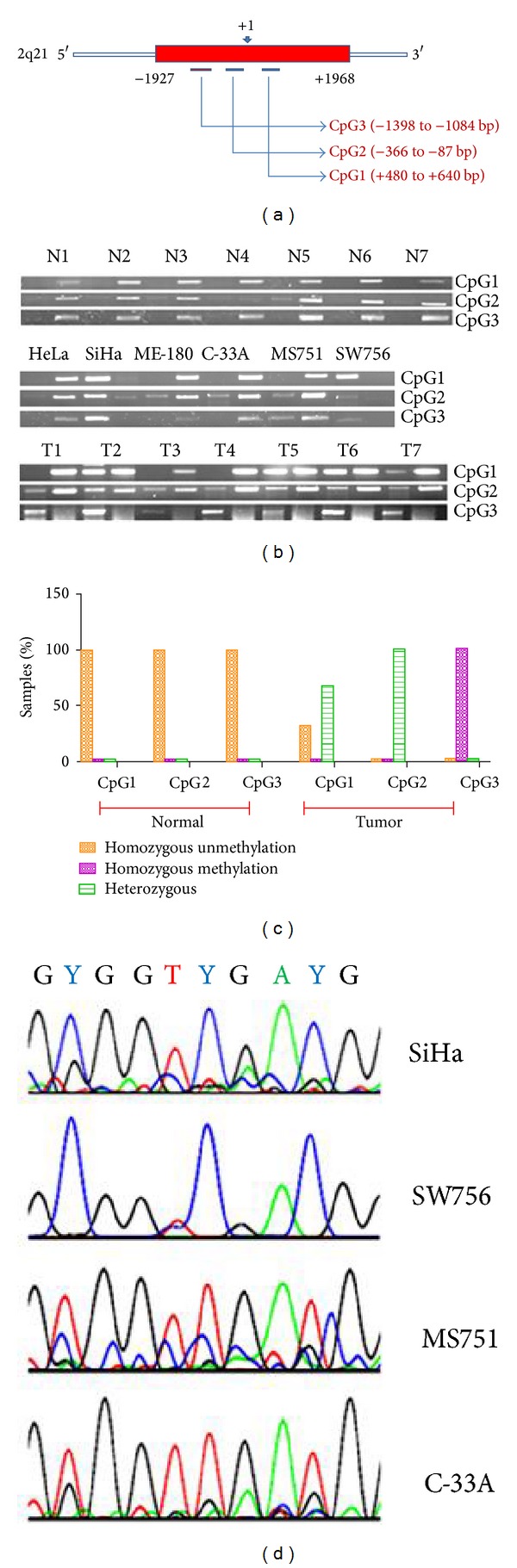
Methylation status of CXCR4 promoter. (a) Cartoon showing CpG island regions and the location of three sets of primers (CpG1, CpG2, and CpG3) from transcription start site (+1). (b) Methylation specific PCR showing the methylation status of three regions of CXCR4 promoter in normal cervix, CC cell lines, and tumor biopsy samples. For each sample shown, first lane is for methylation specific primer set and second lane is for unmethylation specific primer set. N = normal cervix and T = tumor biopsy. (c) Bar diagram showing the summary of methylation status of three CpG regions tested by MSP in normal cervical and tumor biopsies. (d) Representative bisulphite sequencing data of two homozygous methylated (SiHa and SW756) and two homozygous unmethylated (MS751 and C-33A) cell lines showing three CpGs (16th, 17th, and 18th) of CpG1 amplicon. Top panel is the original sequence having these three CpGs where “Y” represents “C” of CpG.

Since CXCR4 expression was found to be downregulated in CC cell lines and primary tumors, we investigated the methylation status of the promoter region of CXCR4. A search for CpG islands using Methyl Primer Express (ABI, USA) in the 5′ region of the CXCR4 gene (gene ID: 7852) revealed two CpG islands (−1922 to +78; %CG = 52.65%, Obs/Exp. CpG > 0.60 and +92 to +1973; %CG = 59.09%, Obs/Exp. CpG > 0.60) that span from the recognized promoter region to the first intron [32]. We selected three regions of the CpG island named as CpG1 (+480 to +640), CpG2 (−366 to −87), and CpG3 (−1398 to −1084) (Figure 2(a)). Our MSP data showed that while the CXCR4 promoter is homozygous unmethylated in normal cervix (*n* = 7) and HeLa cells at all three regions, other cell lines showed either homozygous or heterozygous methylation in at least one of the regions (Figure 2(b) and Table 1). SW756 showed homozygous methylation at all three regions (Figure 2(b) and Table 1). In tumor biopsy samples, CpG1 region was homozygous unmethylated in 32% (*n* = 28) samples and heterozygous in 68% (*n* = 28) samples; CpG2 was heterozygous in all samples and CpG3 was homozygous methylated in all samples (Figures 2(b) and 2(c)). We confirmed the methylation status of CXCR4 promoter and MSP results by bisulfite sequencing PCR (BSP) of CpG1 region in two homozygous methylated (SiHa, SW756) and two homozygous unmethylated (C-33A, MS751) cell lines. BSP showed methylation of all CpGs in SiHa and SW756, while all CpGs of MS751 and C-33A were unmethylated (Figure 2(d)).

### 3.3. Inhibitor of DNA Methyltransferase and Histone Deacetylase Restores the Expression of CXCR4 in CC Cell Lines

**Figure 3:**
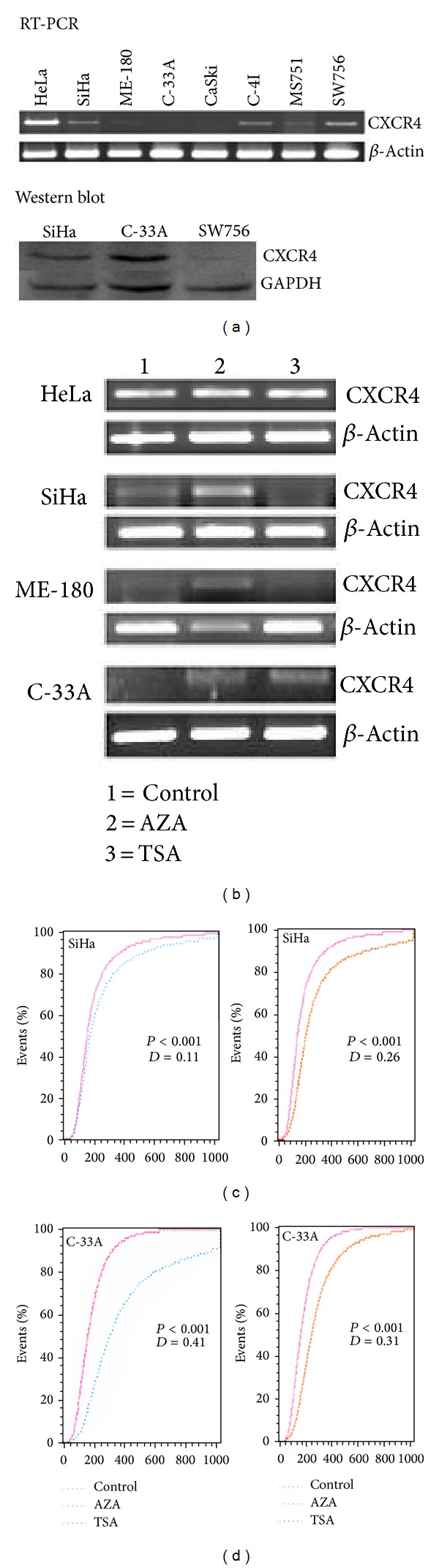
Reactivation of CXCR4. (a) Expression of CXCR4 at transcription (upper panel) and protein level (lower panel) in cervical cancer cell lines. (b) Reactivation of CXCR4 by AZA and/or TSA treatments. Unmethylated HeLa does not show significant change of CXCR4 transcription, and methylated SiHa, ME-180, and C-33A showed reactivation of CXCR4. (c) Fluorocytometry analysis showed reactivation of CXCR4 protein in SiHa after AZA and TSA treatment. (d) Fluorocytometry analysis showed reactivation of CXCR4 protein in C-33A after AZA and TSA treatment. Note the significant reactivation of CXCR4 (maximum vertical deviation between two curves) after the drug treatments. Data were analysed by Kolmogorov Samirnov (KS) test statistics. “*D*” represents the maximum vertical deviation between two curves.

Result of our methylation study showed that all the cell lines except HeLa have either homozygous or heterozygous promoter methylation. Treatment of the cell lines with DNA methyltransferase inhibitor AZA and histone deacetylase inhibitor TSA was done to determine if the transcription of CXCR4 was epigenetically regulated due to DNA methylation or histone deacetylation, respectively. SiHa, ME-180, and C-33A showed reactivation of CXCR4 transcription only after AZA treatment, while C-33A showed reactivation after TSA treatment (Figure 3(b)). This suggests promoter hypermethylation and/or histone deacetylation as one of the major mechanisms of CXCR4 silencing in CC. Similarly, flow cytometry in SiHa and C-33A showed the significant increased expression of CXCR4 protein after AZA and/or TSA treatments (*P* ≤ 0.001; Figures 3(c) and 3(d)). Although SiHa did not show detectable mRNA after TSA treatment, it showed significant increased expression of CXCR4 protein. There was no significant change in the expression of CXCR4 transcript in HeLa, MS751, and SW756 after AZA and/or TSA treatments (Table 1).

### 3.4. Recombinant SDF-1*α* Induces the Cell Adhesion of Cervical Cancer Cells

**Figure 4:**
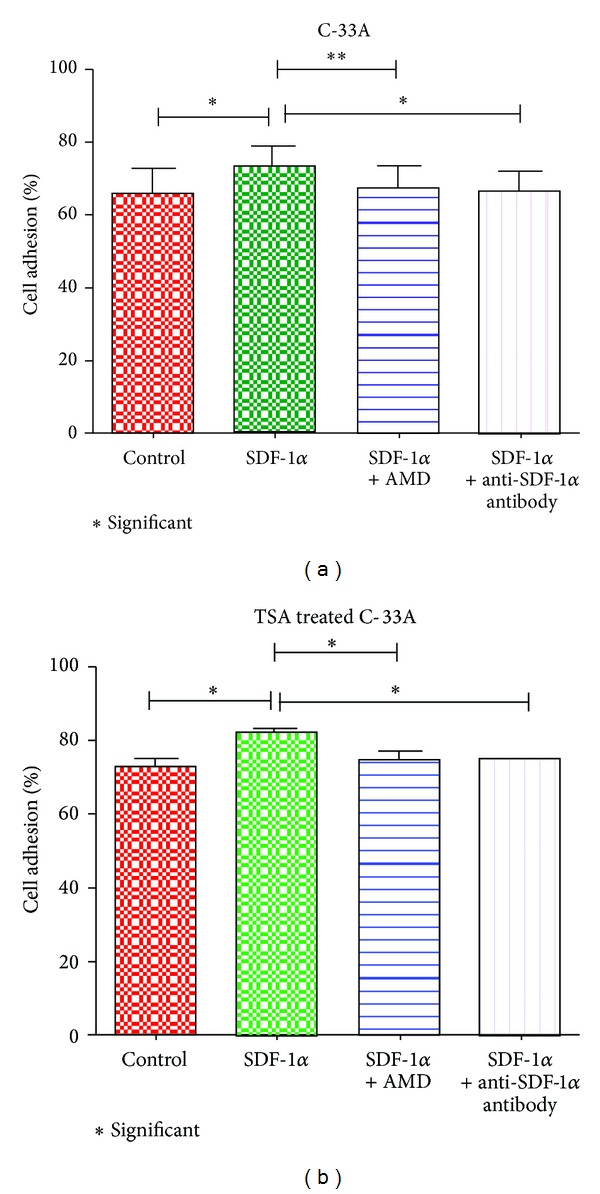
Effect of recombinant SDF-1*α* on C-33A cell adhesion. (a) Adhesion of C-33A cells in presence of recombinant SDF-1*α*. (b) Adhesion of TSA treated C-33A cells in presence of recombinant SDF-1*α*. *P value ≤ 0.05; **P value ≤ 0.005.

In order to observe the effect of SDF-1*α* on adhesion of CC cells to fibronectin coated surface, we did the cell adhesion assay using C-33A in presence of different doses of recombinant SDF-1*α* (5 to 100 ng/mL). Results showed increased cell adhesion in dose dependent manner up to 50 ng/mL (Supplementary Figure S1). To assess the role of SDF-1*α*/CXCR4 signaling in SDF-1*α* induced cell adhesion in C-33A, we evaluated the effect of CXCR4 inhibitor AMD3100 (500 ng/mL) and SDF-1*α* neutralizing antibody on SDF-1*α* induced cell adhesion. SDF-1*α* (50 ng/mL) induced cell adhesion was significantly inhibited in presence of AMD3100 or neutralizing antibody against SDF-1*α* (Figure 4(a)). Similar result was also observed with TSA treated C-33A, but these cells showed enhanced cell adhesion in comparison to untreated cells (Figure 4(b)).

### 3.5. CXCR4 Deficient Cells Do Not Respond to Paracrine Source of SDF-1*α* Leading to the Loss of Cell Adhesion

**Figure 5:**
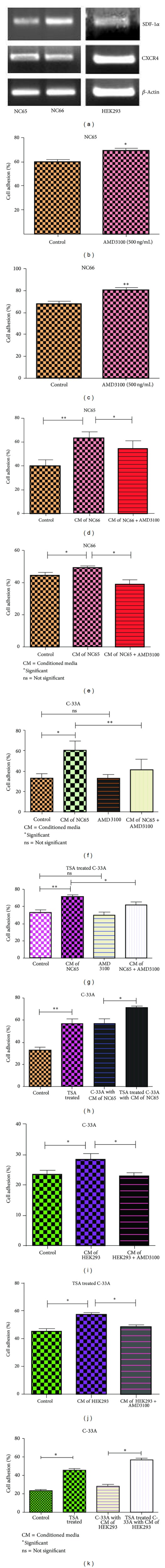
Cell adhesion assay. (a) Expression of CXCR4 and SDF-1*α* in cultured normal cervical cells (NC65 and NC66) and HEK293 by semiquantitative reverse transcriptase PCR; *β*-Actin was used as internal control. (b, c) Adhesion of NC65 and NC66 cells after AMD3100 treatment. (d, e) Adhesion of NC65 and NC66 cells in presence of reciprocal conditioned media. (f, g) Adhesion of untreated and TSA treated C-33A cells in presence of conditioned media of NC65. (h) Comparison of cell adhesion in untreated and TSA treated C-33A cells in presence of conditioned media of NC65. (i, j) Adhesion of untreated and TSA treated C-33A cells in presence of conditioned media of HEK293. (k) Comparison of cell adhesion in untreated and TSA treated C-33A cells in presence of conditioned media of HEK293. *P value ≤ 0.05; **P value ≤ 0.005.

For the cell adhesion experiments, we cultured two normal cervix samples tissues (NC65 and NC66) in RPMI 1640 supplemented with 10% FBS. Our semiquantitative RT-PCR showed the expression of CXCR4 and SDF-1*α* in cultured cells of both the samples (Figure 5(a)). Conditioned media of cells (NC65, NC66, and HEK293) that expressed SDF-1*α* were used as paracrine source of SDF-1*α* for cell adhesion assays. Concentrations of SDF-1*α* in conditioned media of these cells were measured by ELISA which were found to be 4.29 ng/mL, 29.44 ng/mL, and 6.24 ng/mL, respectively. Incubation of the normal cervical (NC65) single cell suspension with AMD3100 (500 ng/mL) for 15 min in fibronectin coated 96-well culture plate significantly induces the cell adhesion (*P* = 0.02; Figure 5(b)) to the fibronectin surface. Similarly, after AMD3100 treatment, cell adhesion of NC66 was also increased (*P* = 0.005; Figure 5(c)). The cell adhesion of NC65 was increased in presence of conditioned media of NC66 (*P* = 0.0007; Figure 5(d)) in comparison to its own conditioned media. AMD3100 treatment inhibited this increased adhesion (*P* = 0.022; Figure 5(d)). Similar result was observed for NC66 cell adhesion in the presence of conditioned media of NC65 (Figure 5(e)). Interestingly, we observed that increase in the cell adhesion of NC65 in presence of conditioned media of NC66 (Figure 5(d)) was more than increase in cell adhesion due to AMD3100 (Figures 5(b) and 5(c)). We performed similar experiments with untreated and TSA treated C-33A using the conditioned media of NC65 (Figures 5(f) and 5(g)) and HEK293 (Figures 5(i) and 5(j)). Note that NC65 and HEK293 both have expression of CXCR4 and SDF-1*α* (Figure 5(a)). The conditioned media of NC65 and HEK293 both significantly induced the cell adhesion of both untreated and TSA treated C-33A cells which was inhibited by AMD3100 (Figures 5(f), 5(g), 5(i), and 5(j)). However, TSA treated cells showed significantly enhanced response to cell adhesion in comparison to untreated cells (Figures 5(h) and 5(k)). Inhibition of autocrine CXCR4 signaling by AMD3100 did not show significant effect on cell adhesion in both untreated (*P* = 0.938; Figure 5(f)) and TSA treated C-33A cells (*P* = 0.525; Figure 5(g)).

## 4. Discussion

In a solid tumor, transformed cells characterized by high genomic instability and altered gene expression are interconnected and communicate with the surrounding microenvironment. The cross talk between microenvironment and cancer cells deeply affects their survival and progression. One of the principle mediators of this cross talk is chemokines. Many lines of evidence showed the involvement of chemokines and their corresponding receptors in cancer progression and metastases [9, 13]. The present study reveals that the transcription of chemokine receptor CXCR4 is epigenetically regulated leading to loss of cell adhesion. It has been previously demonstrated that CXCR4 is frequently upregulated in cancer [13]. However, there is lack of report demonstrating the epigenetic transcriptional regulation of CXCR4 and its consequence in CC progression. Interestingly, our study demonstrates that CXCR4 is frequently downregulated in primary tumors and CC cell lines showed either little or nil expression. Differential downregulation of CXCR4 in tumor biopsy samples indicates antitumor activity of SDF-1*α*/CXCR4 signaling. Similar report also has been demonstrated previously [33]. In addition, our data support the argument that tumors having downregulated CXCR4 may be less responsive to the endogenous and/or exogenous SDF-1*α* compromising the antitumor activity of SDF-1*α*.

The present study demonstrates that downregulated expression of CXCR4 in CC cell lines and primary cervical tumors is due to promoter hypermethylation. Normal biopsy samples showed hypomethylation at promoter regions and normal expression of CXCR4. In tumor biopsy samples, there is no clear negative correlation of promoter methylation with mRNA expression indicating that DNA methylation is not the only mechanism for downregulation of CXCR4 transcription. Among cell lines, only HeLa has the unmethylated promoter and normal expression of CXCR4 while the rest of the cell lines have hypermethylated promoter and showed either little or no expression of CXCR4 suggesting a negative correlation between promoter methylation and expression. Similar negative correlations between CXCR4 expression and promoter methylation have been shown in pancreatic cancer [19] and melanoma cells [34]. Our result is contradictory to the previous report of Łuczak et al. [18] that CXCR4 promoter is not methylated in CC biopsy samples. The dietary intake is known to influence the pattern of global and targeted methylation of genome (reviewed by Alshatwi and Shafi) [35]. They have discussed that dietary factor that modifies global DNA methylation can simultaneously cause opposite effects on gene specific methylation. Vitamin B12 and folate deficiency in general cause the global hypomethylation [36] and may also cause gene specific hypermethylation [37]. Most of the patients included in our study were from states of Uttar Pradesh, Bihar, and Jharkhand state of India. This cohort has the deficiency of both vitamin B12 (49%) and folate (11%) [38] that may lead to gene specific methylation. It also has been reported that 73.9% (*n* = 2668) of people in India have prevalence of vitamin C deficiency [39]. Vitamin C deficiency is associated with DNA hypermethylation in lung cancer cells [40]. In addition, Chung et al. [41] have recently reported that vitamin C mediated DNA demethylation occurs most frequently at CpGs and speculated that vitamin C may indirectly change DNA topology by affecting the histone-acetylation. The above discussion conveys the message that in contrast to other reports the hypermethylation of CXCR4 promoter in our cohort may be the manifestation of inadequate dietary intake of nutritional supplements.

Reactivation of CXCR4 transcription in CC cell lines after DNA methyl transferase (DNMT) inhibitor AZA and/or histone deacetylase inhibitor TSA treatment confirms the epigenetic regulation of CXCR4 and indicates that CXCR4 is epigenetically silenced in CC cell lines. Our flow cytometry experiments in SiHa and C-33A demonstrate that reactivation of CXCR4 transcript is also conveyed to protein level. Reactivation of CXCR4 by AZA and/or TSA has been previously demonstrated also in four pancreatic cancer cell lines (AsPC1, BxPC3, Capan1, Capan2, CFPAC1, and MiaPaCa2) [19] and two breast cancer cell lines (MCF-7 and ZR-75-1) [42, 43]. Other CC cell lines that did not show significant difference after AZA and/or TSA treatments indicate that promoter methylation and histone deacetylation are not the only mechanism regulating the transcription of CXCR4 (Table 1).

**Figure 6:**
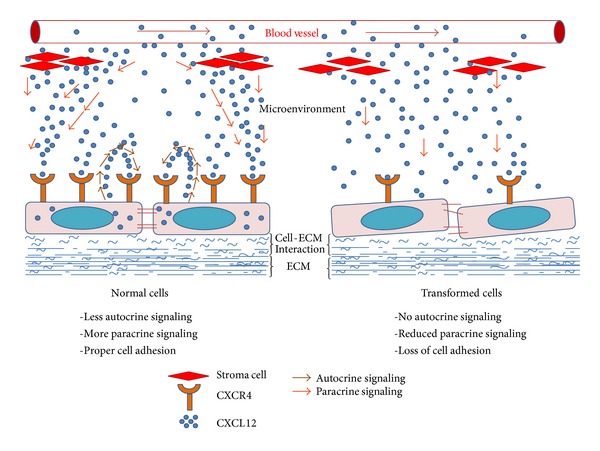
Cartoon showing proposed SDF-1*α*/CXCR4 mediated differential autocrine/paracrine signaling in tumor microenvironment. In normal tissue microenvironment, paracrine signaling dominates over autocrine signaling leading to proper cell adhesion. In tumor (transformed cells) microenvironment, there is reduced paracrine signaling due to reduced level of CXCR4 leading to loss of cell adhesion, where autocrine signaling does not have significant effect.

Differential downregulation of CXCR4 in tumor biopsy and its silencing in CC cell lines prompted us to ask whether autocrine and paracrine SDF-1*α* differentially regulate the CXCR4 signaling in tumor microenvironment. Interestingly, our results demonstrated that downregulation of CXCR4 is responsible for the “loss of adhesion,” one of the most important events responsible for the cancer cell metastases. Inhibition of recombinant SDF-1*α* induced cell adhesion to fibronectin coated surface by application of AMD3100 or anti-SDF-1*α* antibody in C-33A cells indicates that adhesion was mediated by SDF-1*α*/CXCR4 signaling. Similarly, it has been reported that SDF-1*α*/CXCR4 signaling induced cell adhesion of endothelial progenitor cells and colorectal cancer cells [44, 45]. Recombinant SDF-1*α* induced cell adhesion in CC cells may be due to increased expression of cell adhesion molecule ICAM-1, as previously demonstrated by Tung et al. [45] in colon cancer cell lines (DLD-1 and SW48). Inhibition of autocrine SDF-1*α*/CXCR4 signaling by AMD3100 induces cell adhesion in normal cervical cells (NC65 and NC66), suggesting that autocrine SDF-1*α*/CXCR4 signaling positively regulates the loss of cell adhesion. The application of paracrine source of SDF-1*α* (conditioned media of cells having secreted SDF-1*α*, i.e., HEK293 or normal cervical cells NC65 and NC66) also induces cell adhesion suggesting that paracrine SDF-1*α*/CXCR4 signaling negatively regulates the loss of cell adhesion. Blocking the paracrine SDF-1*α*/CXCR4 signaling by application of AMD3100 inhibits the cell adhesion induced by paracrine SDF-1*α*. Hence, this suggests that increased cell adhesion was due to paracrine SDF-1*α*/CXCR4 signaling. Also, cell adhesion was more increased in response to paracrine signaling than autocrine signaling in normal cervical cells. This indicates that the paracrine SDF-1*α*/CXCR4 signaling dominates the autocrine signaling in normal cervical tissue microenvironment. Reduced response to paracrine signaling in NC66 cells was probably due to less expression of SDF-1*α* in NC65 cells than NC66 cells. Our similar sets of cell adhesion assays using C-33A and TSA treated C-33A cells in the presence of conditioned media of NC65 or HEK293 support the observation that paracrine source of SDF-1*α* induces the cell adhesion of CC cells. Response of C-33A to the paracrine source of SDF-1*α* may be due to basal level expression of CXCR4 in C-33A. Further, TSA treated C-33A cells showed enhanced cell adhesion in comparison to C-33A. Addition of AMD3100 to C-33A cells incubated in their own conditioned media did not show significant effect on both untreated C-33A and TSA treated C-33A cells suggesting that autocrine signaling does not play a prominent role in cell adhesion of C-33A. Similar differential impact of autocrine and paracrine SDF-1*α*/CXCR4 signaling in human oral squamous cell carcinoma has been reported by Uchida et al. [46]. In another similar report, Burger et al. [47] have demonstrated that small cell lung carcinoma cells showed induced firm adhesion to M2-10B marrow stromal cells which are considered as the predominant source of SDF-1*α* *in vivo*. Possibly it was due to predominant paracrine SDF-1*α* signaling. Although this study further needs validation in* in vivo* system, based on our current observations, we propose that normal cells having the expression of both CXCR4 and SDF-1*α* predominantly respond to paracrine source of SDF-1*α* resulting in proper cell adhesion, while the transformed cells having reduced expression of CXCR4 do not respond to it, leading to loss of cell adhesion (Figure 6).

## 5. Conclusion

Our results demonstrate that epigenetic silencing of CXCR4 makes the cells inefficient to respond to the paracrine source of SDF-1*α* leading to the loss of cell adhesion, one of the key events involved in initiation of metastases and thereby progression of the disease. Thus, the present study demonstrates antitumor effect of SDF-1*α*/CXCR4 signaling axis involving the maintenance of cell adhesion via paracrine signaling. Based on available data, and to the best of our knowledge, this is the first study in CC showing that CXCR4 is epigenetically downregulated and SDF-1*α*/CXCR4 axis has antitumor activity by maintaining the proper cell adhesion to extracellular matrix. This study also highlights the molecular mechanism of cervical carcinogenesis and importance of the tumor microenvironment in cancer progression.

## Supplementary Material

The primer sequences used in the study is given in supplementary table 1. Fold changes in the expression profile of CXCR4 in the primary tumors compared to normal cervix as shown by real time PCR is given in supplementary table 2. Effect of recombinant SDF-1*α* on adhesion of C-33A cells to fibronectin coated surface is presented in supplementary figure 1.

## Figures and Tables

**Table 1:** Promoter methylation pattern in three regions of CpG islands and reactivation of CXCR4.

Cell lines	Methylation status of CpG1	Methylation status of CpG2	Methylation status of CpG3	Expression (control)	Expression (AZA)	Expression (TSA)	Expression (AZA + TSA)
HeLa	UU	UU	UU	**++++**	**++++**	**++++**	**++++**
SiHa	MM	MU	MM	**+**	**+++**	− − −	**+++**
ME-180	UU	MU	UU	− − −	**+++**	− − −	**+**
C-33A	UU	MU	UU	− − −	**+++**	**+++**	− − −
MS751	UU	MU	MU	**+**	**+**	**+**	**+**
SW756	MM	MM	MM	**+**	**+**	**+**	**+**

MM: homozygous methylated; UU: homozygous unmethylated; MU: heterozygous; ++++: normal expression level.
