# Supplementary material for: Epigenetic Silencing of CXCR4 Promotes Loss of Cell Adhesion in Cervical Cancer
Source: Biomed Res Int. 2014 Jul 10;2014:581403. doi: 10.1155/2014/581403 (PMC4119908; doi:10.1155/2014/581403)

Supplementary Table S1. Nucleotide Sequences of primers used for expression and methylation study

Gene	Expression Primers	
	Forward	Reverse
CXCR4	5'-GGAAAAGATGGGGAGGAGAG-3'	5'- CACTTCCAATTCAGCAAGCA -3'
CXCL12	5'-AGAGCCAACGTCAAGCATCT-3'	5'-GGGCAGCCTTTCTCTTCTTC-3'
βACTIN	5'- AAATCTGGCACCACACCTTC-3'	5'-AGCACAGCCTGGATAGCAAC-3'
	MSP Primers	
CXCR4 (CpG1 Methylation)	5'CGTTCGTTTTAATTTCGGGGTTAAGCG-3'	5'ATCCCCTCAAAAAATCGACGCG3'
CXCR4 (CpG1 Unmethylation)	5'TTGTGTTTGTTTTAATTTTGGGGTTAAGT- 3'	5'-CCCCTCAAAAAATCAACACAATAAA-3'
CXCR4 (CpG2 Methylation)	5'-CGCGTTTTTTATTTGTTTTTAGGC-3'	5'-CTAAAATTTCTAACCGCGACC-3'
CXCR4 (CpG2 Unmethylation)	5'GTTTGTGTTTTTTATTTGTTTTTAGGT-3'	5'-CCACAACCAAACTTTTATAAAAAC-3'
CXCR4 (CpG3 Methylation)	5'-GTTCGTGTCGCGACGTAC-3'	5'-TCCCCATACTCGACGTCTTCCAC-3'
CXCR4 (CpG3 Unmethylation)	5'-GGGGTTTGTGTTGTGATGTAT-3'	5'-AACATCTTCCACAATTTTAAACAAAA-3'
	BSP Primers	
CXCR4	5'TATTTTGTGTATAAGTGTAGAGAAGG-3'	5'-CCTCTACCTACTATACTAAAAA-3'

Supplementary Table S2. Fold change in CXCR4 expression by Real time PCR in cell lines and tumor biopsy samples.

Samples	Fold change	Up/Down Regulated
T8	68.5	Down
T9	32	Down
T10	68.5	Down
T11	16	Down
T12	16	Down
T13	3.03	Down
T14	6.06	Down
T15	1.07	-
T16	10.55	Down
T17	1.4	Down

Supplementary Figure S1: Effect of recombinant SDF-1α on adhesion of C-33A cells to fibronectin coated surface. * P-value ≤ 0.05, ** P-value ≤ 0.005, *** P-value ≤ 0.0005.

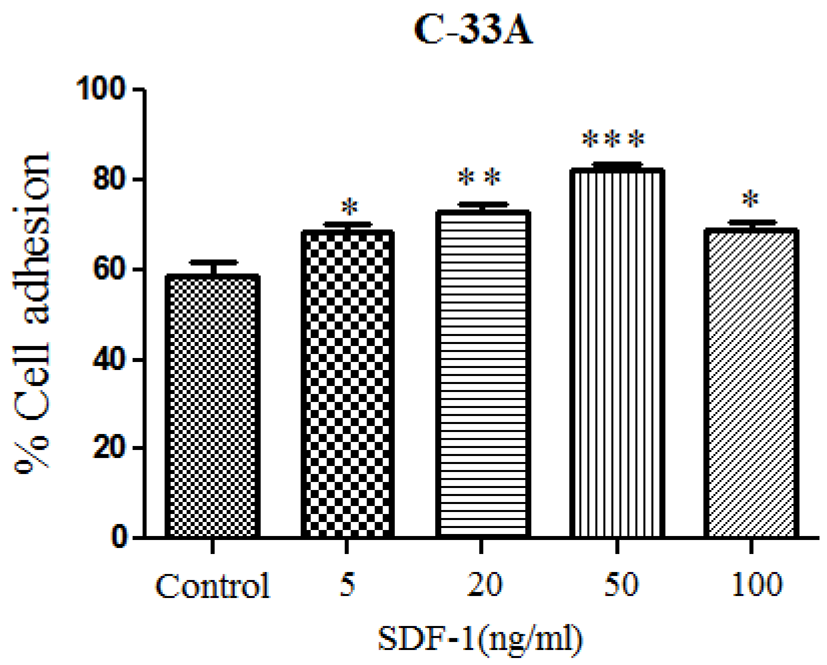

Supplement: Supplementary file 1 — The primer sequences used in the study is given in supplementary table 1. Fold changes in the expression profile of CXCR4 in the primary tumors compared to normal cervix as shown by real time PCR is given in supplementary table 2. Effect of recombinant SDF-1α on adhesion of C-33A cells to fibronectin coated surface is presented in supplementary figure 1. [file 581403.f1.pdf]
